# Different Resistance Exercise Loading Paradigms Similarly Affect Skeletal Muscle Gene Expression Patterns of Myostatin-Related Targets and mTORC1 Signaling Markers

**DOI:** 10.3390/cells12060898

**Published:** 2023-03-15

**Authors:** Mason C. McIntosh, Casey L. Sexton, Joshua S. Godwin, Bradley A. Ruple, J. Max Michel, Daniel L. Plotkin, Tim N. Ziegenfuss, Hector L. Lopez, Ryan Smith, Varun B. Dwaraka, Adam P. Sharples, Vincent J. Dalbo, C. Brooks Mobley, Christopher G. Vann, Michael D. Roberts

**Affiliations:** 1 School of Kinesiology, Auburn University, Auburn, AL 36849, USA; 2 Center for Applied Health Sciences, Canfield, OH 44406, USA; 3 TruDiagnotic, Lexington, KY 40511, USA; 4 Institute for Physical Performance, Norwegian School of Sport Sciences, 0164 Oslo, Norway; 5 School of Health, Medical and Applied Sciences, Central Queensland University, Rockhampton 4700, Australia; 6 Duke Molecular Physiology Institute, Duke University School of Medicine, Durham, NC 03824, USA

**Keywords:** mRNA, protein, acute resistance exercise, gene expression

## Abstract

Although transcriptome profiling has been used in several resistance training studies, the associated analytical approaches seldom provide in-depth information on individual genes linked to skeletal muscle hypertrophy. Therefore, a secondary analysis was performed herein on a muscle transcriptomic dataset we previously published involving trained college-aged men (n = 11) performing two resistance exercise bouts in a randomized and crossover fashion. The lower-load bout (30 Fail) consisted of 8 sets of lower body exercises to volitional fatigue using 30% one-repetition maximum (1 RM) loads, whereas the higher-load bout (80 Fail) consisted of the same exercises using 80% 1 RM loads. Vastus lateralis muscle biopsies were collected prior to (PRE), 3 h, and 6 h after each exercise bout, and 58 genes associated with skeletal muscle hypertrophy were manually interrogated from our prior microarray data. Select targets were further interrogated for associated protein expression and phosphorylation induced-signaling events. Although none of the 58 gene targets demonstrated significant bout x time interactions, ~57% (32 genes) showed a significant main effect of time from PRE to 3 h (15↑ and 17↓, *p* < 0.01), and ~26% (17 genes) showed a significant main effect of time from PRE to 6 h (8↑ and 9↓, *p* < 0.01). Notably, genes associated with the myostatin (9 genes) and mammalian target of rapamycin complex 1 (mTORC1) (9 genes) signaling pathways were most represented. Compared to mTORC1 signaling mRNAs, more MSTN signaling-related mRNAs (7 of 9) were altered post-exercise, regardless of the bout, and *RHEB* was the only mTORC1-associated mRNA that was upregulated following exercise. Phosphorylated (phospho-) p70S6K (Thr389) (*p* = 0.001; PRE to 3 h) and follistatin protein levels (*p* = 0.021; PRE to 6 h) increased post-exercise, regardless of the bout, whereas phospho-AKT (Thr389), phospho-mTOR (Ser2448), and myostatin protein levels remained unaltered. These data continue to suggest that performing resistance exercise to volitional fatigue, regardless of load selection, elicits similar transient mRNA and signaling responses in skeletal muscle. Moreover, these data provide further evidence that the transcriptional regulation of myostatin signaling is an involved mechanism in response to resistance exercise.

## 1. Introduction

Resistance training promotes increases in strength and skeletal muscle hypertrophy [1]. Increasing interest has surrounded how implementing different volume loads affects the cellular responses in the skeletal muscle [2,3,4,5]. Although results from studies examining differences between low and high load resistance training have varied and are difficult to generalize, a relatively consistent theme has emerged suggesting that lower load/higher volume training and higher load/lower volume training promote similar increases in skeletal muscle hypertrophy [4,6]. However, there are unique muscle-molecular differences that have been reported to occur from both forms of training [7]. For instance, evidence exists suggesting lower load/higher volume training elicits more robust mitochondrial adaptations and a greater enhancement in the expression of metabolism-related proteins compared to higher load/lower volume training [8,9,10].

Skeletal muscle molecular responses to resistance exercise are often determined by examining changes in DNA methylation [11,12,13,14], mRNA expression [15,16,17,18,19], and protein expression [8,9,20]. DNA methylation is a mechanism that alters mRNA expression, whereby increased methylation can suppress mRNA expression and suppressed mRNA expression can lead to suppressed protein expression [21]. Our laboratory recently published a report in this special issue investigating how an acute bout of resistance exercise utilizing different volume-load paradigms affected the muscle-molecular milieu [22], specifically mRNA expression and DNA methylation. As we previously reported, the study involved previously trained college-aged males (n = 11; age = 23 ± 4 years old; body mass = 86 ± 12 kg; training experience = 4 ± 3 years) performing two resistance exercise bouts (back squats and leg extensions) at either 30% (30 Fail) of their estimated 1 RM or 80% (80 Fail) of their estimated 1 RM separated by one week. Vastus lateralis muscle biopsies were collected before (PRE), 3 h, and 6 h after each exercise bout, and DNA, along with RNA, were batch-isolated from muscle tissue and analyzed for genome-wide DNA methylation and mRNA expression using the 850 k Illumina MethylationEPIC array and Clariom S mRNA array, respectively. Considerable alterations in both the methylome and transcriptome occurred in the 3 h and 6 h post-exercise period following both the bouts, and bioinformatics analyses indicated that both bouts affected similar pathways (“focal adhesion”, “MAPK signaling”, and “P13K-Akt signaling”). However, the responses between bouts (as determined through bioinformatics) were largely similar. While insights provided through differential expression pipelines are generally useful, it is important to recognize that these applications are more suitable for discovery-based analyses versus mechanistic-based analyses [23,24].

As it relates to the interrogation of genes mechanistically associated with skeletal muscle hypertrophy, existing differential expression pipelines and subsequent pathway analysis tools are limited in their ability to predict a gene’s role in this process. Likewise, gene lists for skeletal muscle hypertrophy (Gene Ontology Consortium; 2021) are perhaps misleading given that genetic manipulation models and other mechanistic preclinical models (e.g., gene therapy and electroporation) have indicated that the knockout, knockdown, or overexpression of dozens of genes alter skeletal muscle mass in adult rodents [23,25,26,27,28,29,30,31,32]. In stark contrast to the underrepresentation of skeletal muscle hypertrophy-associated genes, expansive GOC gene lists of Biological Processes, such as “*Cell Adhesion*” (1500 genes; GO:0098602), “*Cell Cycle*” (1809 genes; GO:0007049), “*Cell Death*” (2237 genes; GO:0008219), and “*Inflammation*” (784 genes; GO:0006954). Accordingly, human resistance exercise research utilizing bioinformatics to interpret acute or longer-term skeletal muscle transcriptomic datasets have generally garnered ambiguous information to the detriment of “drowning out” genes or gene pathways that may be critical to skeletal muscle hypertrophy. This is evident with our own findings suggesting that 30 Fail and 80 Fail exercise bouts acutely altered muscle mRNAs predicted to affect “inflammatory signaling”, “apoptosis signaling”, and “gonadotropin-releasing hormone signaling” [22], while not gaining insight as to whether genes associated with skeletal muscle hypertrophy were affected. Similarly, others have reported that resistance exercise alters genes involved in “macrophage anti-inflammatory polarization” [18] or “stress and cellular compromise, inflammation and immune responses, and necrosis” [33]. It has been recognized that bioinformatics platforms yield limited information on muscle biology and, alternatively, provide pathway and biological process terms that are more relatable to basic cellular biology, cancers, and diseases [34,35,36]. The bias towards alternate biological processes in research conducted using bioinformatics platforms is likely due to the foundational establishment of bioinformatics pipelines and methods being performed by geneticists [37] and cancer biologists [38]. As a result, the information pertaining specifically to skeletal muscle hypertrophy pathways on bioinformatics platforms is underwhelming. 

Herein, we sought to fulfill multiple aims in the current study. First, we examined how 30 Fail versus 80 Fail training affected skeletal muscle mRNAs identified from a literature search that have been linked to skeletal muscle mass regulation using preclinical models [23,25,26]. Further, we interrogated protein and phosphorylated-protein markers of select targets to examine the downstream implications of our transcriptomic analysis. Due to the exploratory nature of this study, we adopted a null hypothesis for all aims herein. 

## 2. Methods

### 2.1. Participants and Ethical Approval

This study was conducted with prior review and approval from the Auburn University Institutional Review Board and in accordance with the most recent revisions of the Declaration of Helsinki (IRB approval #: 20-081 MR 2003). Raw DNA methylation data and mRNA data can be found in the Gene Expression Omnibus (www.ncbi.nlm.nih.gov/geo/; GEO accession numbers: GSE220928 for DNA methylation data (public on 14 December 2022), and GSE220899 for mRNA array data (scheduled for release on 1 June 2023)).

College-aged males (n = 11) were recruited from the local community, and all were required to have participated in lower-body training at least once per week over the last 6 months. Other details regarding inclusion and exclusion criteria can be found in the parent publication by Sexton et al. [22].

### 2.2. Study Design

A more comprehensive description of the study design and methodology can be found in Sexton et al. [22]. Briefly, a crossover study design was implemented that included a total of five laboratory visits. Informed consent and screening forms were completed at visit 1, and following these procedures, participants performed maximal strength testing on the barbell-back and knee extension exercises. Visit 2 consisted of vastus lateralis (VL) muscle biopsies being collected from participants prior to completion of an exercise bout consisting of four sets of back squats to failure and four sets of leg extensions to volitional failure using a randomly assigned experimental load (30% or 80% estimated 1 RM). VL muscle biopsies were collected at both 3 h and 6 h, respectively following exercise during visit 2. Participants returned 7 days following visit 2 and performed the same training bout with the load that was not allocated during visit 2, and VL biopsies were collected at the PRE, 3 h, and 6 h timepoints as well. Visits 3 and 5 were biopsy checks to ensure that wounds were healing properly. It is finally worth noting that participants completed training bouts following an overnight fast between the hours of 0700 and 1000 and that exercise bouts were completed at similarly scheduled times by each participant. 

### 2.3. Wet Laboratory Analyses

*Muscle tissue processing for simultaneous DNA, RNA, and protein isolation.* In-depth details regarding tissue processing can be found by Sexton et al. [22]. Briefly, skeletal muscle samples (15–20 mg) were homogenized using Trizol and bromochloropropane (BCP) (instead of chloroform). Centrifugation divided the sample into an aqueous phase containing RNA, DNA interphase, and a bottom protein phase. A portion of the aqueous phase was removed and processed to yield RNA, which was sent to a commercial laboratory for gene expression analysis. The remaining DNA and protein were then separated by biochemical and centrifugation procedures. This produced a DNA pellet and a protein supernatant, and the protein-Trizol-ethanol supernatant was removed for protein isolation. Following various biochemical steps described by Sexton et al., the resultant protein pellet was resuspended in 2× sodium dodecyl sulfate (SDS) sample loading buffer + 5M Urea (1:2 dilution of 10 M Urea, 2:5 dilution of 5× SDS sample loading buffer, 1:10 deionized water, and 1:100 50× protease inhibitor). A commercial assay (RC DC Protein Assay, catalog #5000122; Bio-Rad; Hercules, CA, USA) was used to quantify protein content, and samples were diluted to a standardized concentration using deionized water and stored at −80 °C until Western blotting. 

*mRNA and DNA methylation arrays.* Isolated RNA was shipped to a commercial laboratory for transcriptomic analysis (North American Genomics, Decatur, GA, USA). Following the quantification of gene expression using the Clariom S Assay_Human mRNA array. Raw.CEL files were uploaded into the Transcriptome Analysis Console v4.0.2 (TAC) (Thermo Fisher Scientific, Waltham, MA, USA) and subsequently annotated via the *h. sapiens* genome (Hg 38 build). Data were then normalized using the robust multiarray average (RMA) normalization method and are presented as log_2_-signal intensity. 

Isolated DNA was shipped to a commercial laboratory (TruDiagnostic, Lexington, KY, USA), and methylation analysis was performed using the Infinium MethylationEpic BeadChip Array (Illumina; San Diego, CA, USA) per the manufacturer’s instructions. Additional details regarding data analysis can be found in Sexton et al. [22]. These data were manually interrogated for DNA methylation results regarding hypertrophy-related genes.

*Western blotting.* Protein samples that were prepared as discussed above were loaded onto 4–15% SDS-polyacrylamide gels (Bio-Rad) and subjected to 50 min of electrophoresis (180 V) using SDS-PAGE running buffer (VWR). Proteins were transferred to polyvinylidene difluoride (PVDF) membranes (Bio-Rad) using a wet blotting apparatus at 200 mA for 2 h. Following transfers, PVDF membranes were Ponceau stained and imaged in a gel documentation system (ChemiDoc Touch, Bio-Rad, Hercules, CA, USA) to ensure equal protein loading between lanes. Following 1 h blocking at room temperature with 5% nonfat milk powder in Tris-buffered saline with 0.1% Tween-20 (TBST; VWR), membranes were incubated overnight (pan proteins) or for ~48 h (phospho-proteins) with the following antibodies at a 1:1000 dilution in TBST: (i) rabbit anti-human phospho-mTOR (Ser2448; Cell Signaling, Cat #: 5536), (ii) rabbit anti-human pan mTOR (Cell Signaling, cat #: 2983), (iii) rabbit anti-human p-p70S6K (Thr389; Cell Signaling, cat #: 2983), (iv) rabbit anti-human pan p70S6K (Cell Signaling, cat #: 9202), (v) rabbit anti-human phospho-AKT (Ser473; Cell Signaling, cat #: 4060), (vi) rabbit anti-human pan AKT (Cell Signaling, catalog #: 4691), (vii) mouse anti-human follistatin (FST; Life Technologies Corporation, Carlsbad, CA, USA, cat #: 60060-1), and (viii) mouse anti-human myostatin (MSTN; Life Technologies Corporation, catalog #: MA531804). Following primary antibody incubation periods, membranes were incubated with horseradish peroxidase-conjugated anti-rabbit or anti-mouse antibodies (1:2000; Cell Signaling) in TBST with 5% BSA at room temperature for 1 h. An enhanced chemiluminescent reagent (Luminata Forte HRP substrate; Millipore Sigma, Burlington, MA, USA) was used to develop membranes in a dark gel documentation station (ChemiDoc Touch, Bio-Rad), and band densitometry was performed using associated software. Densitometry values of FST and MSTN were normalized to Ponceau densities that corresponded to ~25–75 kD. Phosphorylated target band densities were divided by the pan densities of respective targets to obtain a ratio. All data are expressed as relative expression units.

*Literature review to identify candidate genes mechanistically associated with skeletal muscle hypertrophy.* Our strategy for identifying candidate genes mechanistically associated with skeletal muscle hypertrophy was three-fold. First, the five genes provided by GOC (GO: 0014734) were included in our gene target list (*AR, IGFBP5, MTOR, MYMK,* and *MYOC*). Next, a systematic literature review published in 2018 by Verbrugge and colleagues provided a list of 47 additional genes from mechanistic preclinical studies in which the knockout, knockdown, or overexpression of these genes were found to increase skeletal muscle weight or myofiber cross-sectional area [23]. Finally, we performed a literature review from May 2018 to August 2022 using the search terms “muscle hypertrophy” and “knockout”, “knockdown”, or “overexpression” to identify another seven genes, including *UBR5* [26], *RPTOR* [27], *TRIM28* [28], *MAPK8/JNK1* [29], *PSMC4* [30], *ATG7* [31], and *YAP1* [32]. The final 58 genes selected for interrogation were *TPT1*, *GNAS*, *PPARGC1A*, *CAST*, *ASB15*, *MCU*, *BMPR1A*, *NR3C1*, *THRA*, *SMAD4*, *KLF10*, *DGAT1*, *RHEB*, *NR4A1*, *IGF1*, *NCOR1*, *MSTN*, *MAPK8*, *PSMC4*, *YAP1*, *IKBKB*, *UBR5*, *NOL3*, *GRB10*, *TRIM28*, *EIF2B5*, *PPARD*, *XIAP*, *ATG7*, *AKT1*, *AGTR1*, *DGKZ*, *LTBP4*, *PLD1*, *ACVR2B*, *RPTOR*, *ESR1*, *TP53INP2*, *CRTC2*, *ADRB2*, *JUNB*, *INHBB*, *SKI*, *INHBA*, *FST*, *GPRASP1*, *FSTL3*, *MMP9*, *CAMKK1*, *PAPPA*, *CRYM*, *BDKRB2*, *UCN3*, *AR*, *IGFBP5*, *MTOR*, *MYMK*, and *MYOC*.

### 2.4. Statistics

As described by Sexton et al. [22] Transcriptome Analysis Console v4.0.2 (TAC) (Thermo Fisher Scientific) was used to analyze all mRNA data. Specifically, two-way repeated measure (2 × 2) ANOVAs were used to identify potential interactions between bouts for each of the 58 candidate mRNAs. Main time effects or interactions were considered significant if *p* < 0.01 and if the expression at PRE did not differ between conditions (using a *p*-value threshold of less than 0.05). All Western blot data were analyzed with SPSS (v29.0; IBM Statistics, Chicago, IL, USA) using two-way within-within repeated measures ANOVAs, and LSD post hoc tests were used to decompose the main effects of time. All data in tables and figures are presented as mean ± standard deviation (SD) values, and, in all cases except methylation data, individual respondent data are also presented. 

## 3. Results

### 3.1. mRNA Expression following Acute Bouts of Resistance Exercise

**Figure 1:**
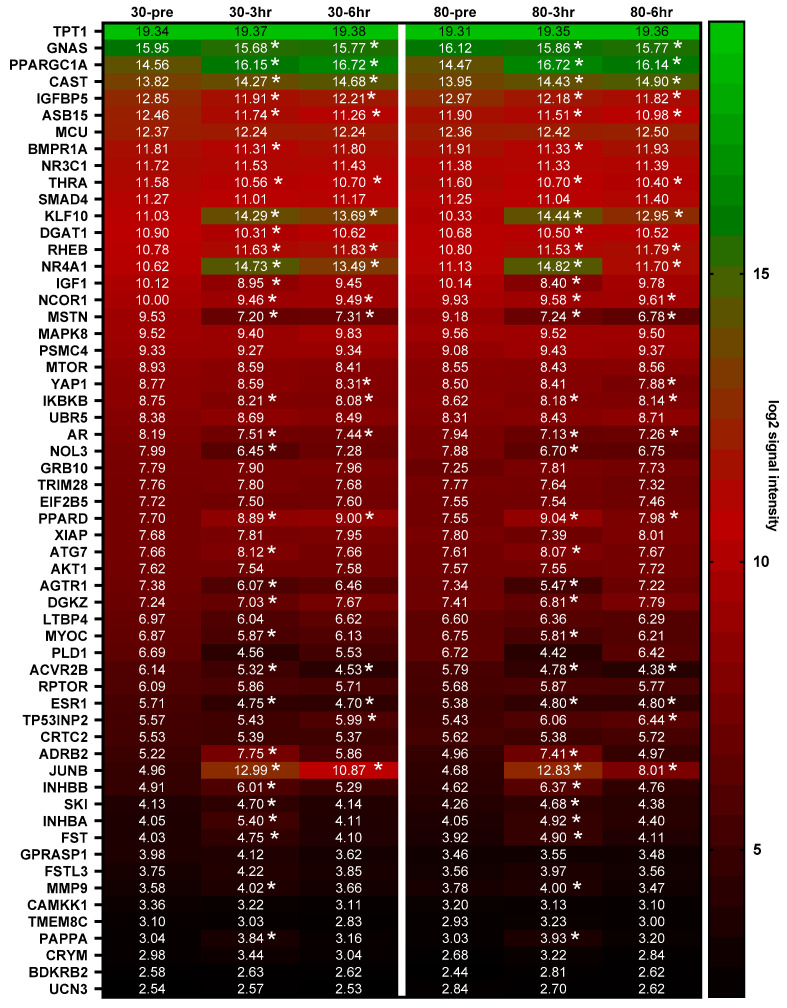
Heat map of mRNAs related to hypertrophy and their acute responses to the 30 Fail or 80 Fail bouts. These data show the log_2_ expression of the 58 mRNAs related to skeletal muscle hypertrophy. Genes are sorted (top to bottom) based on basal expression at the 30 Fail PRE condition. Notably, only significant time effects (not interaction effects) were evident (denoted by *, *p* < 0.01).

Figure 1 depicts all mRNAs of candidate genes identified to be mechanistically associated with skeletal muscle hypertrophy. There were no significant interactions; however, ~57% of these mRNAs showed a main effect of time from PRE to 3 h (15↑ and 17↓, *p* < 0.01), and ~26% showed a main effect of time from PRE to 6 h (8↑ and 9↓, *p* < 0.01). 

### 3.2. Myostatin-Associated Gene Responses

Genes involved with MSTN signaling (9 genes: *ACVR2B, BMPR1A, FST, FSTL3, INHBA, INHBB, MSTN, SKI, SMAD4*) made up a large portion of the 58 genes interrogated. Notably, 7 of these 9 genes (all except *SMAD4* and *FSTL3*) displayed a main effect of time. Given that several of these mRNAs were dynamically altered, we had access to the methylation data of these genes from Sexton et al. [22], we also examined how various CpG sites were affected in tandem with mRNA expression patterns. 

**Figure 2:**
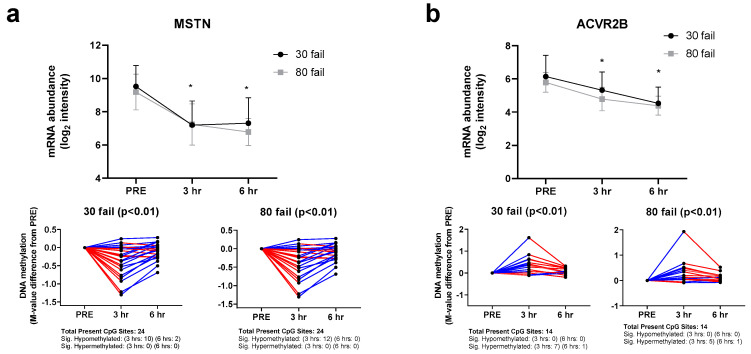
MSTN and ACVR2B mRNA and DNA methylation responses. mRNA and methylation responses of myostatin (MSTN, panel (**a**)) and Activin A Receptor Type 2B (ACVR2B, panel (**b**)), which is the cognate receptor for myostatin. Notably, resistance exercise (regardless of load) downregulated these mRNAs, whereas only ACVR2B showed several CpG sites being significantly hypermethylated by resistance exercise. Symbol: *, indicates mRNA at 3 h or 6 h post-exercise was significantly downregulated. Other note: CpG sites are depicted as M-value changes from PRE, where red coloration indicates a directional change downward, and blue indicates a directional change upward.

*MSTN* and *ACVR2B* regulation at the DNA methylation and mRNA expression level is illustrated in Figure 2a,b. Notably, both were downregulated at the mRNA level following 30 Fail and 80 Fail resistance exercise bouts. The respective subpanels illustrate how CpG sites associated with these genes responded to each form of resistance exercise. For *MSTN*, of the 24 associated CpG sites investigated, none showed significant hypermethylation at 3 h or 6 h, indicating that the downregulation in *MSTN* was likely unrelated to DNA methylation events. Conversely, of the 14 *ACVR2B*-associated CpG sites investigated, several showed significant hypermethylation at 3 h, indicating that the downregulation in *ACVR2B* may have been related to DNA methylation events.

**Figure 3:**
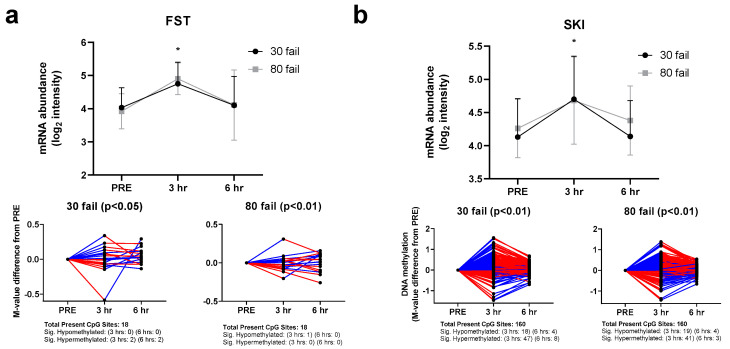
FST and SKI mRNA and DNA methylation responses. mRNA and methylation responses of follistatin (FST, panel (**a**)) and SKI Proto-Oncogene (SKI, panel (**b**)), both of which are inhibitors of myostatin signaling. Notably, resistance exercise (regardless of load) upregulated these mRNAs, and neither mRNA response was seemingly associated with CpG site methylation patterns. Symbol: *, indicates mRNA at 3 h or 6 h post-exercise was significantly downregulated. Other note: CpG sites are depicted as M-value changes from PRE, where red coloration indicates a directional change downward, and blue indicates a directional change upward.

*FST* and *SKI* regulation at the DNA methylation and mRNA expression level is illustrated in Figure 3a,b. Both genes were upregulated at the mRNA level following 30 Fail and 80 Fail resistance exercise bouts. The respective subpanels illustrate how CpG sites associated with these genes reacted to each form of resistance exercise. For *FST*, most of the 18 *FST*-associated CpG sites investigated (16 during 30 Fail and 17 during 80 Fail) did not show significant alterations in methylation status at 3 h or 6 h, indicating that the upregulation in *FST* was likely unrelated to DNA methylation events. Alternatively, of the 160 SKI-associated CpG sites investigated, several (77 during 30 Fail and 67 during 80 Fail) showed significant paradoxical methylation at 3 h, indicating that the upregulation in *SKI* was also unlikely unrelated to DNA methylation events.

### 3.3. mTORC1-Associated Gene Responses

Nine genes that are part of the mTORC1 signaling cascade *(AKT1, DGKΖ, EIF2B5, IGF1, IGFBP5, MTOR, PLD1, RHEB, RAPTOR)* also made up a large portion of the 58 genes interrogated. Of these genes, *RHEB* (up at 3 h and 6 h, *p* < 0.001 and *p* < 0.001) was the only one that was upregulated. Genes involved with mTORC1 signaling that were downregulated included *IGFBP5* (down at 3 h and 6 h, *p* < 0.001 and *p* < 0.001) and *DGKZ* (down at 3 h, *p* = 0.004). 

### 3.4. Western Blot Results for AKT-mTOR, Myostatin and Follistatin Protein Targets

**Figure 4:**
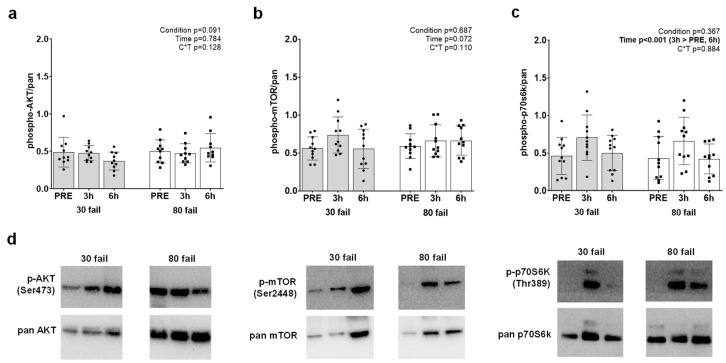
Phospho-signaling responses of select AKT-mTORC1 targets. Phosphorylation responses of AKT (panel (**a**)), mTOR (panel (**b**)), and p70S6K (panel (**c**)), both of which are inhibitors of myostatin signaling. Notably, resistance exercise (regardless of load) increased the phosphorylation status of p70S6K. Panel (**d**) contains representative Western blots of the assayed targets.

Given that gene targets involved with MSTN and mTORC1 signaling were highly prevalent on our list of 58 genes, we opted to perform Western blotting on these targets. AKT-mTORC1 pathway proteins are presented in Figure 4. While no significant interactions were identified, and no significant group or time effects were evident for phospho- (Ser473)/pan AKT (Figure 4a) or phospho- (Ser2448)/pan mTOR (Figure 4b). There were, however, increases in phospho- (Thr389)/pan p70S6K (PRE to 3 h, *p* = 0.001; Figure 4c). 

**Figure 5:**
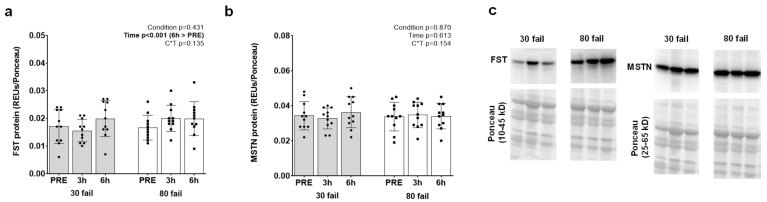
FST and MSTN protein responses. The effects of each bout on the protein expression of follistatin (FST) (Panel (**a**)) and myostatin (MSTN) (Panel (**b**)) are presented. Resistance exercise (re-gardless of load) increased FST levels 6 h following exercise. Panel (**c**) contains representative blots of the assayed targets; note these targets came from the same participant, which is why the Ponceau stains are similar.

Protein abundance data for follistatin and myostatin proteins are presented in Figure 5. A significant main effect of time was evident for follistatin protein abundance (PRE to 6 h, *p* < 0.001; Figure 5a), albeit a significant interaction was not evident. No significant interaction or main effects were evident for MSTN protein levels (Figure 5b).

## 4. Discussion

This investigation aimed to extend the findings reported by Sexton et al. [22] by determining differences in the mRNA expression of 58 genes associated with skeletal muscle hypertrophy following acute bouts of 30 Fail and 80 Fail resistance exercise. Although our data suggest both modes of exercise elicited similar mRNA expression profiles, several MSTN-related mRNAs were dynamically altered regardless of load. Both bouts also similarly affected mTORC1 signaling mRNAs and upregulated p70S6K phosphorylation following exercise. Finally, both bouts transiently elevated follistatin protein levels while not affecting myostatin protein levels. Our findings provide additional insight into the molecular responses to lower versus higher load resistance exercise to failure.

Among the genes identified during our literature review, as well as those identified by Verbrugge et al. [23], nine of them belonged to the myostatin signaling pathway (*ACVR2B, BMPR1A, FST, FSTL3, INHBA, INHBB, MSTN, SKI, SMAD4*) [23]. *MSTN* has been demonstrated as having a role in the suppression of the skeletal muscle growth [39], possibly through inhibition of the mTORC1 [40]. The *ACVR2B* gene encodes for a receptor that mediates MSTN signaling through phosphorylation (i.e., activation) of SMAD transcription factors [41]. *FST* is essential for muscle fiber formation and growth by inhibiting the myostatin binding [42]. *FSTL3* similarly inhibits myostatin and activin A, which suppresses their functionality and promotes muscle growth [43,44]. *INHBA* is a muscle growth inhibitor and encodes for activins which are a part of the TGF-β superfamily and have roles in the development regulation [45,46]. *INHBB* is also a part of the TGF-β superfamily and is a muscle growth inhibitor [47,48]. *SKI* also impairs *MSTN* signaling due to its ability to inhibit the activity of the SMADs [49,50], and *SMAD4* acts as an intracellular mediator of TGF-β signaling [51]. Seven of these MSTN-related genes exhibited significant mRNA expression responses along with dynamic changes in methylation statuses. Moreover, there was generally a downregulation in mRNA targets that potentiate MSTN signaling (e.g., *MSTN* and *ACVR2B*), whereas there was an up-regulation in mRNA targets that inhibit MSTN pathway activation (e.g., *FST* and *SKI*). These findings exhibit similarities and differences with past reports in the literature. For instance, multiple studies have reported that skeletal muscle *MSTN* mRNA is downregulated in response to acute and longer-term resistance training periods [52,53,54,55]. Others have reported skeletal muscle *ACVR2B* mRNA expression in older adults (age = 68 ± 6 years) is not altered, while another study has reported increased *ACVR2B* mRNA expression in younger recreational athletes (age = 27 ± 5 years) rehabilitating from ACL surgery [56,57], and *FST* mRNA expression has been shown not to be affected in skeletal muscle following one and multiple sessions of resistance exercise [58,59]. Indeed, differences in participant demographics and study designs (e.g., exercises and load prescriptions) are likely responsible for the observed differences between studies. Notwithstanding, our data suggest multiple targets of the MSTN pathway, regardless of load selection, appear to be transiently regulated at the mRNA level in response to resistance exercise. A notable finding herein was that, while several MSTN inhibitor mRNAs and follistatin protein levels were up-regulated and MSTN mRNA was downregulated in the post-exercise period, MSTN protein levels remained unaltered. In attempting to reconcile these findings, potential explanations exist. First, the upregulation in MSTN inhibitors likely reduced MSTN signaling itself (i.e., SMAD2/3 phosphorylation) rather than acted upon MSTN protein levels. Indeed, we attempted to blot for SMAD2/3 phosphorylation but did not obtain a sufficient signal across membranes to quantify results. Hence, while some evidence exists in this area [56], more research examining SMAD2/3 phosphorylation (and nuclear localization) following acute resistance exercise bouts is needed. Regarding MSTN protein levels remaining unaltered in the post-exercise period in lieu of down-regulated *MSTN* mRNA levels, this was likely due to biopsy sampling being too early to detect a downregulation in protein levels. In this regard, others have shown that immediate post-exercise alterations in PGC1-α mRNA levels lead to protein expression changes that are measurable ~24 h following the exercise bout [60]. Thus, again, more research is needed to time course MSTN mRNA and protein-level changes following exercise, given that this gene is highly responsive to resistance exercise.

Nine genes involved with mTORC1 signaling (*AKT1, DGKΖ, EIF2B5, IGF1, IGFBP5, MTOR, PLD1, RHEB, RPTOR*) were also included in the current analysis. Briefly, the mTORC1 pathway is considered an anabolic signaling hub, and its activation in skeletal muscle involves an upregulation of several signaling proteins by way of phosphorylation which leads to increased translation initiation resulting in increased muscle protein synthesis [61,62]. *AKT1* encodes a kinase that acts to activate the mTORC1 protein kinase [63]. *DGKΖ* encodes for an enzyme that acts to increase cellular phosphatidic acid levels in response to mechanical overload, and phosphatidic acid binds to mTOR, resulting in mTOR activation [64]. *IGFBP5* is a binding protein that transports insulin-like growth factor variants in circulation and modulates ligand effectiveness [65]. *EIF2B5* encodes for a translation initiation factor which acts to increase rates of translation initiation and, subsequently, protein synthesis [66,67]. *IGF1* encodes for a growth factor that supports muscle growth by activating the tyrosine kinase IGF1 receptor, thus stimulating PI3K-AKT signaling [68,69]. *RPTOR* encodes a protein that binds to and upregulates the mTORC1 signaling [27,70], and *RHEB* encodes for a GTPase that stimulates the mTORC1 activity [71]. While many of the myostatin pathway-related genes displayed dynamic alterations to the acute bouts of training, the mTORC1 signaling-related mRNAs were not as responsive or were paradoxically affected following exercise. Specifically, *RHEB* mRNA increased during the post-exercise period, *DGKZ*, *IGF1,* and *IGFBP5* mRNAs were downregulated, and no changes were observed for *AKT1, EIF2B5, MTOR, PLD1,* or *RPTOR*. These findings both agree and disagree with prior findings in the literature. For instance, resistance exercise has been reported to transiently up-regulate *IGF1* mRNA in the post-exercise period [72], which counters our findings. Although reasons for this discrepancy are difficult to posit, it likely has to do with these prior studies examining untrained participants versus the trained participants who were examined herein. However, our *RHEB* findings agree with Wang et al. [73] reported an increase in this mRNA 1 h following acute resistance exercise in recreationally trained participants (age = 26 ± 1.2 years). Similarly, our *IGFBP5* mRNA data agree with a report by Dennis et al. [74], who demonstrated that this mRNA is downregulated 72 h following an acute bout of resistance exercise in younger adults. Our *MTOR* mRNA findings also align with data published by Drummond et al. [75] showed *MTOR* mRNA was not affected 3 h following resistance exercise. When considering the collective evidence, it appears that certain genes critical to mTORC1 signaling (e.g., *MTOR* and *RPTOR*) may not be transcriptionally responsive to resistance exercise, whereas RHEB is upregulated, and this may be a transcriptional mechanism involved with the hypertrophic response to resistance exercise.

Given that our gene list (Figure 1) contained several mTORC1 gene targets, we opted to perform Western blotting on associated phosphorylated proteins [76,77,78,79,80,81,82]. Three mTORC1 signaling proteins were selected for immunoblotting analysis (phosphorylated [Thr389]/pan AKT, phosphorylated [Thr389]/pan p70S6K, phosphorylated/pan mTOR [phosphorylated at Ser2448]). Notably, Mitchell et al. [4] and Haun et al. [83] are the only two prior studies that have examined mTORC1 signaling markers following a lower-load and higher-load resistance exercise bout. Phosphorylated AKT was not affected between time points, which agrees with the findings of Mitchell and colleagues, who reported that the post-exercise phosphorylation status of this protein did not differ when participants performed three sets of leg extensors to failure using 30% or 80% 1-RM loads. Phosphorylated p70S6K was upregulated 3 h following an acute bout of resistance exercise, which agrees with prior data from our laboratory [83] demonstrating that p70S6k phosphorylation is increased 15 and 90 min following higher (80% 1 RM) and lower load (30% 1 RM) leg extensor resistance exercise to volitional fatigue. We observed no changes in phosphorylated mTOR protein levels following exercise, which again agrees with the findings reported by Haun et al. [83], albeit, disagrees with the findings of Mitchell et al. [4]. Despite the minor discrepancies between the current study and the two prior investigations, the collective data continue to suggest that post-exercise mTORC1 signaling differences do not seemingly exist between lower-load and higher-load bouts of resistance exercise so long as each set is executed to volitional fatigue.

In addition to the AKT-mTOR pathway protein targets, two myostatin pathway protein targets (myostatin and follistatin) were interrogated, given the robust changes we observed in MSTN-related mRNAs in response to both modes of resistance exercise. The interrogation of these targets with the current study design is novel given that they were not investigated in the 30 Fail versus 80 Fail Mitchell et al. [4] or Haun et al. [83] studies. Notably, we observed no changes in myostatin protein expression, and this agrees, in part, with a report by Snijders et al. [84], who demonstrated that myostatin protein levels remain unaltered in skeletal muscle at early post-exercise time points and become down-regulated 72-h following resistance exercise. We also observed an increase in follistatin protein 6-h following exercise, regardless of load. Although resistance exercise literature examining this muscle protein is sparse, this finding agrees, in principle, with other studies reporting that circulating follistatin concentrations increase during resistance training interventions [85,86]. Hence, in lieu of the mRNA data discussed above, these data suggest that a downregulation in *MSTN* mRNA in response to resistance exercise may eventually matriculate into a decrease in muscle protein levels days following the bout, as discussed above. Conversely, the rapid post-exercise increase in *FST* mRNA levels may result in protein levels increasing soon thereafter.

## 5. Conclusions

Pathway coverage of genes mechanistically associated with skeletal muscle hypertrophy is underrepresented in traditional pipelines used to elucidate differential gene expression. When considering the collected data, it is apparent that a bout of lower and higher load training to failure similarly affects the 58 interrogated mRNAs mechanistically associated with skeletal muscle hypertrophy. Further, select mTORC1 signaling markers are similarly affected between bouts, as is the protein expression of follistatin. Critically, our secondary analysis provides in-depth information regarding how the mRNA expression of genes mechanistically associated with skeletal muscle hypertrophy was affected following two unique resistance exercise bouts to failure. Regarding investigations seeking to elucidate underlying mechanisms of muscle growth, we recommend that researchers adopt a targeted approach with directed attention to gene expression analysis.

## Data Availability

Queries related to these data can be addressed by C.G.C. (christopher.vann@duke.edu) and M.D.R. (mdr0024@auburn.edu), and raw data will be provided by M.D.R. upon reasonable request.
